# Small RNAs and extracellular vesicles: New mechanisms of cross-species communication and innovative tools for disease control

**DOI:** 10.1371/journal.ppat.1008090

**Published:** 2019-12-30

**Authors:** Qiang Cai, Baoye He, Arne Weiberg, Amy H. Buck, Hailing Jin

**Affiliations:** 1 Department of Microbiology and Plant Pathology, Center for Plant Cell Biology, Institute for Integrative Genome Biology, University of California, Riverside, California, United States of America; 2 Department of Biology, Ludwig-Maximilians University of Munich (LMU), Munich, Germany; 3 Institute of Immunology and Infection Research, School of Biological Sciences, University of Edinburgh, Edinburgh, United Kingdom; 4 Centre for Immunity, Infection and Evolution, School of Biological Sciences, University of Edinburgh, Edinburgh, United Kingdom; THE SAINSBURY LABORATORY, UNITED KINGDOM

## Overview

Small RNA (sRNA)-mediated RNA interference (RNAi) is a conserved regulatory mechanism for gene expression throughout the domain Eukarya. Recent studies have shown that sRNAs can move between a host and an interacting organism to induce gene silencing in *trans*, a mechanism termed “Cross-Species RNAi” or, in many cases, “Cross-Kingdom RNAi.” Pathogens and parasites transport sRNAs into host cells during infection and silence host defense genes to suppress immunity, whereas hosts can also deliver their sRNAs into interacting microbes or parasites to suppress infection. Recent studies of different plant and animal hosts and their interacting organisms have unveiled extracellular vesicles (EVs) as vehicles of sRNA exchange in cross-species and cross-kingdom RNAi. The discovery of the pivotal role of sRNAs and EVs in cross-species and cross-kingdom communication offers innovative tools for pathogen and pest control in agriculture and biomedicine.

## Cross-kingdom RNAi

**Fig 1:**
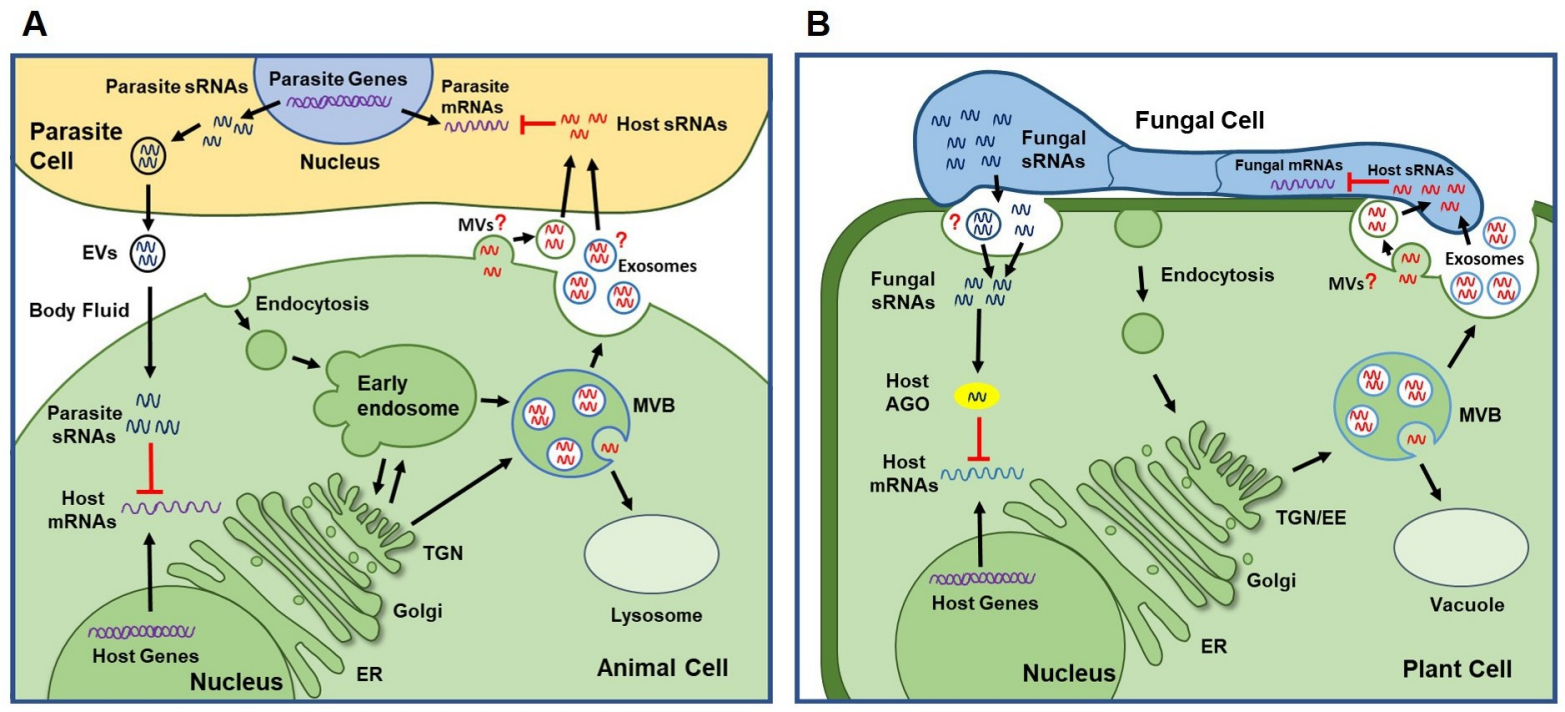
Cross-species and cross-kingdom RNAi between host and coinhabitants. (A) Cross-species RNAi between mammals and parasites. Parasites produce EVs containing parasitic sRNAs, which are internalized by mammalian cells to silence host genes involved in inflammation and innate immunity. Animal cells can deliver sRNAs into interacting organisms. They also secrete EVs (e.g., exosomes or MVs) containing host sRNAs. It is likely that animal hosts may also transport sRNAs using EVs into parasites to suppress parasitic genes. (B) Cross-kingdom RNAi between plants and fungal pathogens. Fungal sRNAs translocate into plant cells and hijack host AGO protein of the RNAi machinery to suppress plant immune responses. It is still unclear how pathogens transport sRNAs. Conversely, plants secrete EVs to transport host sRNAs into pathogens to silence fungal genes involved in virulence. The “?” indicates a prediction that has not been validated experimentally. AGO, Argonaute; EE, early endosome; ER, endoplasmic reticulum; EV, extracellular vesicle; MV, microvesicle; MVB, multivesicular body; RNAi, RNA interference; sRNA, small RNA; TGN, trans-Golgi network.

sRNAs—including microRNAs (miRNAs) that are processed by Dicer-like (DCL) proteins from single-stranded stem-loop–forming RNA precursors and small interfering RNAs (siRNAs) that are processed by DCL proteins from double-stranded RNA (dsRNA) precursors—are loaded into Argonaute (AGO) proteins to induce silencing of genes with complementary sequences [1]. Some sRNAs from diverse classes of pathogens and parasites are transported into host cells and induce cross-kingdom or cross-species RNA silencing to facilitate infection (Fig 1). Fungal pathogens, including ascomycete and basidiomycete species, can deliver sRNAs into their respective hosts [2–6]. In detail, *Botrytis cinerea*, the grey mold fungal pathogen that infects over 1,000 plant species, delivers sRNAs into plant cells and hijacks host RNAi machinery by loading its sRNAs into the *Arabidopsis* AGO1 protein to trigger silencing of host immunity genes, including mitogen-activated protein kinases (MAPKs), cell-wall–associated kinases, and other defense and signaling proteins [2]. A panel of sRNAs from *Verticillium dahliae*, which causes *Verticillium* wilt in many plant hosts, also move into plant cells and associate with the host AGO1 protein to silence host genes involved in plant defense [4]. A genome-wide association study shows that the white mold fungal pathogen *Sclerotinia sclerotiorum* produces sRNAs that, to facilitate infection, can target plant genes associated with quantitative disease resistance [5]. A miRNA-like sRNA from *Puccinia striiformis*, the causal agent of the destructive wheat stripe rust, targets wheat pathogenesis-related genes and suppresses host immunity to achieve successful infection [3]. Likewise, the parasitic plant *Cuscuta campestris* (dodder) transports several miRNAs into *A*. *thaliana* and *Nicotiana benthamiana* to promote invasion [7].

Cross-kingdom sRNA trafficking from a fungal pathogen to an animal host was also observed recently. *Beauveria bassiana*, an insect fungal pathogen, exports a miRNA-like RNA (bba-milR1) to the host mosquito, which induces cross-kingdom RNAi to suppress host immunity [6]. Strikingly, this insect fungal pathogen-derived bba-milR1 also binds to host AGO1 and silences mosquito target gene Toll receptor ligand Spätzle 4 [6], which is consistent with the mechanism used by transported sRNAs from plant fungal pathogens [2, 4].

In addition to eukaryotic pathogens, prokaryotic microbes can also use cross-kingdom RNA trafficking to manipulate gene expression in the hosts. Specifically, the root-nodule bacterium *Rhizobium* delivers tRNA-derived sRNA fragments (tRFs) into soybeans to suppress host genes involved in nodule formation and root development, which enhances nodulation efficiency [8]. Surprisingly, these *Rhizobium* tRFs also function through host AGO1 [8], just like fungal pathogen-derived sRNAs that are bound with host AGO1 to silence host target genes [2, 4, 6]. Furthermore, it has long been known that virus- or viroid-derived sRNAs can target various host protein-coding genes to facilitate infection in both plant and animal hosts [9–14]. A recent study revealed that the targeting of a long noncoding RNA in tomato by tomato yellow leaf curl virus-derived sRNAs contributes to disease symptoms [15].

Cross-kingdom RNAi is bidirectional. Plant hosts also transport sRNAs into fungal pathogens to suppress the expression of virulence-related genes, which contributes to plant defense responses. Translocation of plant endogenous sRNAs into fungi was clearly demonstrated by sRNA profiling of fungal cells purified from infected plant tissue [16]. Cai and colleagues developed an innovative sequential protoplastation method, which allowed for the removal of all plant cells and the purification of *B*. *cinerea* protoplasts/cells from infected *Arabidopsis* tissue [17]. These purified fungal cells contain host miRNAs and siRNAs, including *Trans*-acting siRNAs, also called secondary phasing siRNAs (phasiRNAs) [16]. These *Arabidopsis* sRNAs are delivered into interacting *B*. *cinerea* cells to induce silencing of fungal genes that are involved in pathogenicity, many of which are related to vesicle trafficking [16]. Mutated *B*. *cinerea* strains with a deletion in these target genes displayed reduced pathogenicity on plant hosts [16]. Another study found that cotton miRNA166 and miRNA159 accumulated in the mycelium of *V*. *dahliae* grown on artificial agar medium 30 days post re-isolation from infected tissue, which suggests that cotton miRNAs can translocate into *V*. *dahliae* [18]. Both cotton miRNAs trigger silencing of *V*. *dahliae* genes involved in virulence, *Ca*^*2+*^*-dependent cysteine protease* (*Clp-1*), and *isotrichodermin C-15 hydroxylase* (*HiC-15*), which enhances disease resistance against this vascular pathogen [18]. Similarly, the wheat miRNA1023 suppresses an *alpha/beta hydrolase* gene in *Fusarium graminearum*, which is important for fungal infection [19]. Plant sRNA-induced silencing of pathogen genes is not restricted to fungi. A similar phenomenon was later observed in the interaction between plants and an oomycete pathogen, *Phytophthora capsici*. *Arabidopsis* may use secondary sRNAs to silence *Phytophthora* genes during infection [20].

Cross-species RNAi also exists in animal–parasite interactions. Some mammalian parasites use cross-species RNAi strategies to silence host genes and enable infection. For instance, the gastrointestinal nematode *Heligmosomoides polygyrus* (also known as *H*. *bakeri*) secretes sRNAs, including miRNAs, which suppress type II innate immune response in the murine host [21]. Conversely, some animal hosts also deliver sRNAs into parasites. Patients who suffer from sickle cell anemia show abnormal erythrocyte development but exhibit resistance to the malaria parasite *Plasmodium falciparum*. One of the reasons for malaria resistance is that these patients accumulate higher levels of a specific panel of miRNAs, which are transported into the parasite and suppress *P*. *falciparum* virulence [22]. Though *P*. *falciparum* lacks canonical RNAi components, such as DCLs and AGOs, the authors demonstrated that cross-kingdom RNA regulation occurs through impaired ribosomal loading by the fusion of host miRNAs with the parasite target mRNAs. This chimerization blocks target mRNA translation and causes an inhibition of parasite growth [22]. Anti-*Plasmodium* cross-kingdom RNA regulation was also reported based on the human miR-451/140 targeting the *P*. *falciparum antigen erythrocyte membrane protein-1* (*PfEMP1*). Human miR451 was found in the parasitic cell in complex with human AGO2, providing the first example of cross-kingdom delivery of an sRNA–AGO complex [23].

In the mammalian gut, miRNAs secreted by human and mouse intestinal epithelial cells were shown to influence gene expression even in gut bacteria that lack canonical RNAi machinery, suggesting a regulatory role of host miRNAs in gut microbiome homeostasis [24]. Furthermore, dietary plant miRNAs can also enter gut bacteria through plant-derived exosome-like nanoparticles, further shaping the gut microbial community [25]. RNAi does not exist in prokaryotes per se; however, bacteria have various ribonucleases, including type III ribonucleases [26], which may interact with the host or dietary miRNAs to interfere with bacterial mRNA expression. The increasing number of discovered cases of cross-species and cross-kingdom RNAi or RNA *Trans*-regulation across diverse host–microbe and host–parasite systems has made it clear that cross-species and cross-kingdom RNA communication is likely a ubiquitous mechanism in host–microbe and host–parasite interactions.

## EVs in animal–parasite interactions

In mammals, RNAs circulating through body fluids are often encapsulated in extracellular vesicles (EVs). EVs are membrane-surrounded vesicular compartments released by cells to the extracellular environment to transport proteins, RNAs, lipids, and other molecules to other cells or to interacting organisms [27]. EVs are categorized into multiple classes based on their biogenesis pathways and associated protein markers. In mammalian systems, multiple classes of EVs have been shown to carry sRNAs. In particular, exosomes, which are derived from multivesicular bodies (MVBs) and have tetraspanin proteins as one of the key protein markers [28], play an important role in sRNA trafficking [29]. Microvesicles, which bud from the plasma membrane, can also transport sRNAs into recipient cells [30]. Both types of EVs are involved in cell-to-cell communication in homeostasis, immune signaling, and neural networks [31, 32]. While exosomes and microvesicles are secreted during normal cellular processes, apoptotic bodies are formed during programmed cell death [33]. Functional molecules, including RNAs, can be detected in apoptotic bodies [34, 35]. Some reports have shown that apoptotic bodies can transport these functional molecules into recipient cells [35, 36], though whether they are also involved in cross-kingdom communication between parasites/microbes and animal hosts remains to be explored.

It is not surprising that pathogens and pests would evolve to exploit or target these natural cell-to-cell communication pathways. Diverse parasites have been shown to use EVs to deliver sRNAs to host cells and modulate host gene expression (Fig 1A) [37]. The miRNA-containing EVs that are released by the gastrointestinal nematode—or helminth—*H*. *polygyrus* are internalized by host mouse cells and suppress inflammation and innate immune responses during infection [21]. Many of the nematode miRNAs share common ancestry and identical seed sites with miRNAs of the mouse host, such that they would be expected to be able to tap into existing miRNA target networks in the mouse cell. However, the RNAi mechanisms used between these two animals are complex, as the nematode packages a nematode-specific AGO protein (extra cellular worm Argonautes [exWAGO]) into the EVs bound to siRNAs from rapidly evolving nongenic regions of the parasite genome [38]. Indeed, these studies suggest that different parasites and pathogens might have diverse tools for RNA-mediated suppression of host genes. The study of these pathogen RNA transmission mechanisms may guide new strategies for effective therapeutic delivery of RNAs (for example, delivering RNA–AGO complexes, rather than RNA alone) [39]. Since the EVs from helminths are immune suppressive, the EVs and their RNA cargoes also represent another potential therapy for treating colitis and allergies in humans [21, 40, 41].

In mammalian systems, EVs have been shown to transport sRNAs between cells within the organism; we speculate that EVs may also be used by the host cells to deliver sRNAs to its interacting organisms, such as parasites and pathogens.

## EVs in plant–microbe interactions

In 1967, plant EVs were initially observed in carrots by electron microscopy [42]. Forty years later, Regente and colleagues isolated plant EVs from extracellular wash fluids of imbibed sunflower seeds [43]. However, the origin of plant EVs still remained unknown. In mammals, exosomes are a class of EVs derived from MVBs. Mammalian tetraspanins cluster of differentiation (CD)63, CD81, and CD9 are enriched in exosome membranes and are commonly used as biomarkers to isolate and phenotype exosomes [28]. *Arabidopsis* encodes 17 members of the *TETRASPANIN* (*TET*) family [44], and two *Arabidopsis TET*s (*TET8* and *TET9*) are induced upon infection by *B*. *cinerea*. Moreover, TET8-associated vesicles accumulated to a high level at the fungal infection sites [16]. TET8 is colocalized with *Arabidopsis* MVB-marker Rab5-like GTPase ARA6 inside the cell, and TET8-associated vesicles are secreted into the apoplast [16], suggesting that TET8-associated EVs are derived from MVBs and secreted into apoplastic space and can, therefore, be considered bona fide plant exosomes. These exosomes contain plant-endogenous sRNAs and are efficiently taken up by *B*. *cinerea* fungal cells. Plant exosomes deliver sRNAs into fungal pathogens to suppress fungal infection by inducing silencing of fungal virulence-related genes. Similarly, *Arabidopsis* also transports secondary phasiRNAs from *PPR* gene clusters into an oomycete pathogen, *P*. *capsici*, likely also by EVs, which silence target genes in the pathogen [20]. Thus, plants have adapted EV-mediated cross-kingdom RNAi for immune responses during the coevolutionary arms race with interacting pathogens (Fig 1B).

In addition to exosomes, PENETRATION (PEN)1-associated EVs, which contain several stress-response–related proteins, were identified in *Arabidopsis* [45]. The biogenesis pathway of PEN1-associated EVs remains unclear, although PEN1 was originally identified as a plasma-membrane–associated plant-specific syntaxin [46]. PEN1-associated EVs were purified from the apoplast wash fluid of *Arabidopsis* leaves using an ultracentrifugation speed (40,000*g*) [45, 47], which is slower than that used to isolate TET-associated exosomes (100,000*g*) [16]. Secretion of PEN1-assoiated EVs was increased during infection by a bacterial pathogen (*Pseudomonas syringae*) or following treatment with the phytohormone salicylic acid [45]. Baldrich and colleagues analyzed the sRNA population in these EVs isolated from uninfected *Arabidopsis* leaves and found that these EVs carry predominantly “tiny RNAs,” which are 10–17 nucleotides in length and derived mainly from the positive strand of mRNA transcripts [48]. It is not clear whether these tiny RNAs have any biological function. Since pathogen-infected samples were not included in this study, whether this class of EVs is also involved in plant and pathogen interactions and whether tiny RNAs are delivered into pathogen cells via these EVs remain unclear. PEN1 and the ATP-binding cassette (ABC)-transporter PEN3 are incorporated into extracellular encasements surrounding the haustoria of the powdery mildew fungus, *Golovinomyces orontii*, suggesting that PEN1-asociated EVs contribute to defense responses against powdery mildew [45, 49, 50]. A third type of plant EV, which is derived from a novel double-membrane–bound exocyst-positive organelle (EXPO) [51], has been reported in plants. These EXPO-derived EVs were discovered through transient expression of exocyst subunit exo70 family protein E2 (Exo70E2), a component of exocyst complex, in protoplasts from *Arabidopsis* suspension-cultured cells. Whether EXPO-derived EVs contain RNAs and are involved in cross-kingdom communication remains to be discovered.

Similar to animal EVs, which comprise diverse, heterogeneous, and cell-type–specific populations with a wide range of biological functions in cell-to-cell communication [52], the previously cited studies suggest that plant cells also secrete different classes of EVs that may contain specific cargoes. Establishing plant EV biomarkers (such as TET8, PEN1, and Exo70E2) will enable immuno-based analysis of EVs to further understand the biological functions of EVs in complex biological systems such as plant–microbe interactions.

Though EV-mediated transport is a key mechanism for RNA secretion and delivery between hosts and microbes/pests, nonvesicular extracellular RNAs have also been discovered. Specifically, in human plasma, extracellular RNAs were found within RNA–protein complexes, including AGO proteins and high-density lipoprotein complexes [53–56]. Additionally, exomeres, extracellular nonmembranous nanoparticles, have recently been discovered in mammalian systems containing AGO1, AGO2, and AGO3 proteins; amyloid precursor proteins; RNAs; and DNAs. Notably, these exomeres contained a profile of macromolecules distinct from exosomes [57, 58]. In a plant system, Baldrich and colleagues found that sRNAs were still present in apoplastic wash fluid, which they believe was depleted of EVs by centrifugation at 40,000*g* [48]. However, small, RNA-containing EVs, such as exosomes, are mostly collected at higher speeds (between 100,000*g* and 120,000*g*) from various plant and mammalian systems [52, 59–61], as well as from fungi [62, 63]. Furthermore, plant tetraspanin-labeled exosomes, which transport sRNAs from the hosts to fungal cells, were much more enriched after centrifugation at a speed of 100,000*g* than at 40,000*g* [16]. Therefore, it is unlikely that plant EVs can be depleted at 40,000*g*, and, consequently, whether nonvesicular RNAs are secreted by plants requires further investigation. Furthermore, the origins of nonvesicular RNAs and their potential role in cross-kingdom RNAi remain to be explored.

## RNA and EV-based innovative tools for disease control

Global disease control mainly relies on chemical protection measures using fungicides, pesticides, and antibiotics, which not only threatens the health of humans and ecosystems but also generates novel uncontrollable drug-resistant pathogenic strains [64]. We are in urgent need of innovative, durable, and eco-friendly fungicides and antimicrobial drugs to avoid a global collapse in our ability to control pathogen/parasite infections in both plants and animals, including humans.

One direct application of cross-kingdom RNAi is host-induced gene silencing (HIGS), a promising technology in which transgenic plants express dsRNAs or sRNAs that target pathogen or insect virulence-related genes to combat plant diseases [65, 66]. This approach has also made it possible to control multiple pathogens spontaneously by designing dsRNA and sRNA constructs that target multiple genes from different pathogens [4]. Although HIGS is effective, it involves the generation of genetically modified organisms (GMOs), which is not only technically challenging in many crop species but unfortunately still a concern for many consumers. Furthermore, GMOs are banned in European agricultural productions, rendering HIGS not practically usable, at least in the near future.

**Fig 2:**
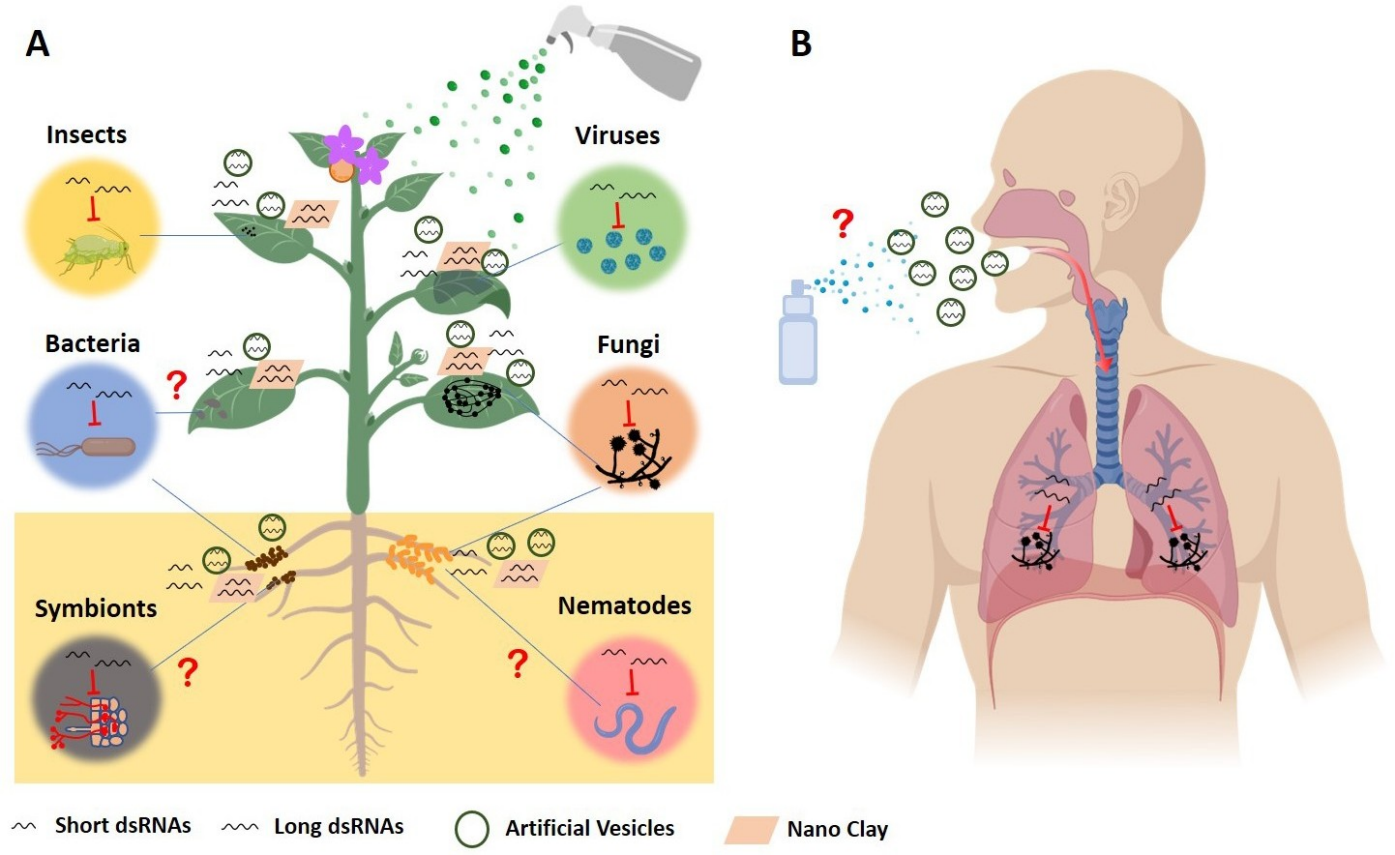
SIGS is an efficient disease control strategy in plants and potentially in humans. (A) Spray application of dsRNAs and sRNAs that target pathogen/pest genes can potentially control plant diseases. The SIGS-based protection can be prolonged by incorporating RNAs into artificial vesicles (black circle) or nanoparticles (pink rhombus) to protect RNAs from degradation or water rinsing. (B) Future RNA-based antifungal drugs have the potential to control human mycoses. Artificial vesicles/liposomes will likely facilitate the RNA delivery. Figures were created with BioRender. The “?” indicates a prediction that has not yet been validated experimentally. dsRNA, double-stranded RNA; SIGS, spray-induced gene silencing; sRNA, small RNA.

Environmental RNAi, initially discovered in the nematode *Caenorhabditis elegans* [67], is the cellular uptake of RNAs from the environment and the induction and spreading of systemic gene silencing. Forward genetics screening in *C*. *elegans* revealed that *Systemic RNA interference deficient* (*SID*)*-1* and *SID-2* encode for two dsRNA transmembrane channel proteins, which are required for dsRNA uptake and systemic gene silencing [68, 69]. In this invertebrate system, there is higher uptake and silencing efficiency for long dsRNA (>60 bp) than short (<25 bp) or single-stranded RNA [70, 71]. Inspired by environmental RNAi of *C*. *elegans*, Wang and colleagues tested whether fungal cells can also take up RNAs from the environment and observed rapid RNA uptake by *B*. *cinerea* cells [4]. These RNAs induce silencing of fungal genes in a sequence-specific manner. Unlike *C*. *elegans*, which primarily takes up long dsRNAs, fungal uptake of environmental RNAs seems less dependent on RNA size, because both short sRNA duplexes and long dsRNAs are taken up by fungi and induce robust gene silencing in the fungal cells [4]. Fungal environmental RNAi allowed plant scientists to design spray-induced gene silencing (SIGS) to control fungal and potentially other pathogens through spray application of pathogen gene-targeting dsRNAs and sRNAs (Fig 2A) [4, 72, 73]. Wang and colleagues demonstrated that spray application of long dsRNAs or sRNA duplexes that target *B*. *cinerea DCL1* and *DCL2* genes can effectively suppress grey mold diseases on fruits, vegetables, and flowers [4]. Koch and colleagues have shown that SIGS can also effectively control a fungal disease in the monocot barley [73]. Spray application of a long dsRNA that targets fungal cytochrome P450 *lanosterol C-14α-demethylase* genes on barley leaves can inhibit *F*. *graminearum* infection [73]. Similarly, application of exogenous dsRNAs helps protect *Brassica napus* from infection by *S*. *sclerotiorum* and *B*. *cinerea* [74]. These pathogen gene-targeting dsRNAs and sRNAs represent a novel class of eco-friendly fungicides, “RNA fungicides” (Fig 2A).

Exogenous RNAs can either be directly internalized into fungal cells [4] or indirectly via passage through plant tissue before transport into interacting pathogen cells [75]. Furthermore, Koch and colleagues observed inhibition of *F*. *graminearum* growth in the distal nonsprayed barley leaf tissue [73], suggesting that sprayed dsRNAs taken up by plant cells moved through vasculature systemically. While the molecular mechanism of RNA uptake in *C*. *elegans* and some nematodes is based on SID proteins, which are not present in plants or fungi, the mechanisms for uptake of environmental RNAs into fungi and plants need further investigation.

Obviously, the effectiveness of SIGS relies on extracellular RNA stability and RNA uptake efficiency of pathogens. To technically improve RNA stability, Mitter and colleagues docked an antiviral dsRNA onto double hydroxide clay nanosheets, which increased the efficacy of plant antiviral protection [75]. In addition, the use of artificial vesicles or liposomes to protect RNAs could be an effective strategy to improve SIGS for plant protection and to develop potential antifungal drugs for therapy, as some fungi are capable of taking up EVs efficiently (Fig 2) [16, 76]. Since EV trafficking is also a natural RNA transport mechanism in mammals, it is exciting to consider the potential for extension of artificial vesicle-protected RNA-based antifungal strategies in humans (Fig 2B). Indeed, lipid-based nanoparticles have been used to stabilize therapeutic compounds, including sRNAs, in biomedical applications [77]. For example, liposomal amphotericin B, the world’s leading antifungal drug, was based on liposomal formulation of amphotericin B to reduce toxicity [78]. Moreover, Walker and colleagues have observed that amphotericin B–containing liposomes remained intact during transit through the cell walls of phylogenetically distant fungal pathogens, *Candida albicans* and *Cryptococcus neoformans*, although liposomes (60–80 nm) are larger than the theoretical cell wall porosity (approximately 5.8 nm)[79]. This work suggests that the fungal cell wall is deformable and viscoelastic to allow liposomes to pass, which makes it possible to efficiently deliver new generation of antifungal drugs, including RNA-based drugs, using liposomes/artificial vesicles [79]. In 2018, the Food and Drug Administration (FDA) approved the very first therapeutic siRNA drug, patisiran, to treat hereditary transthyretin-mediated amyloidosis, a rare, debilitating and often fatal genetic disease [80]. Patisiran uses a lipid nanoparticle delivery mechanism to transfer 21-bp siRNA duplex into cells in the liver [80]. Besides patisiran, there are at least 6 other RNAi therapeutics already in phase III clinical trials [80].

Although more than 300 human or animal pathogenic fungal species have been recorded and fungal infections display disproportional high mortality rates, mycoses are rather neglected in infection biology research [81]. Survival rates of patients suffering from respiratory and systemic fungal infections often caused by the opportunistic fungi *Candida* (candidiasis), *Aspergillus* (aspergillosis), or *Cryptococcus* (cryptococcosis) are low due to limited availability of antifungal drugs. Drug-resistant fungal strains have already emerged to all the commonly used antifungal drugs [64]. Therefore, innovative drugs to combat fungal infections are urgently needed, and based on the effects observed for antifungal SIGS approaches in plants, development of novel antifungal RNA therapeutics and artificial vesicle/liposome-mediated delivery methods may be effective in the fight against mycoses.

## Future perspectives

The field of cross-species and cross-kingdom communication via RNA is still in its infancy, yet an increasing number of studies across diverse systems demonstrate that mobile RNAs are key regulatory molecules that shape the interactions between hosts and interacting pathogens or organisms. Plants and animals deliver sRNAs into interacting (micro-)organisms to inhibit infection, and pathogens and parasites can, in turn, transport sRNAs into the host to suppress host immunity. Current studies show that EVs play an essential role in transporting sRNAs from the plant hosts to pathogens and from parasitic nematodes to mammalian hosts, and it is very likely that mammalian hosts could also utilize EVs to deliver sRNAs into their parasites and pathogens, though this is currently just speculation. Recent advances in methodology development for isolating different classes of EVs in mammalian systems provide excellent tools and guidelines to study RNA delivery in cross-species and cross-kingdom RNAi [82, 83]. Although there is diversity in the properties of EVs based on their cell and tissue origin (and purification techniques, which can also impact the exact profile of RNAs and proteins found in EVs), it is clear that small EVs, including exosomes, play an important role in delivering sRNAs [60, 84, 85].

The discovery of cross-species and cross-kingdom RNAi and fungal RNA uptake has inspired scientists to design novel disease control strategies against pathogens and pests in agriculture, such as HIGS and SIGS. Structural and mechanistic studies of EVs in sRNA trafficking allows for the development of innovative delivery methods of sRNAs using artificial vesicles, or nanoparticles, which may also be considered for therapeutic applications in mammalian systems. We speculate that future development and application of a new generation of RNA-based fungicides and antifungal drugs will be an important research direction to control diseases caused by eukaryotic pathogens and parasites.

## References

[1] Baulcombe D. RNA silencing in plants. Nature. 2004;431: 356–363. 10.1038/nature02874 15372043

[2] Weiberg A, Wang M, Lin FM, Zhao H, Zhang Z, Kaloshian I, et al Fungal small RNAs suppress plant immunity by hijacking host RNA interference pathways. Science. 2013;342: 118–123. 10.1126/science.1239705 24092744 PMC4096153

[3] Wang B, Sun YF, Song N, Zhao MX, Liu R, Feng H, et al *Puccinia striiformis f*. *sp tritici* microRNA-like RNA 1 (*Pst-milR1*), an important pathogenicity factor of *Pst*, impairs wheat resistance to *Pst* by suppressing the wheat pathogenesis-related 2 gene. New Phytol. 2017;215: 338–350. 10.1111/nph.14577 28464281

[4] Wang M, Weiberg A, Lin FM, Thomma BP, Huang HD, Jin H. Bidirectional cross-kingdom RNAi and fungal uptake of external RNAs confer plant protection. Nat Plants. 2016;2: 16151 10.1038/nplants.2016.151 27643635 PMC5040644

[5] Derbyshire M, Mbengue M, Barascud M, Navaud O, Raffaele S. Small RNAs from the plant pathogenic fungus *Sclerotinia sclerotiorum* highlight host candidate genes associated with quantitative disease resistance. Mol Plant Pathol. 2019;20: 1279–1297. 10.1111/mpp.12841 31361080 PMC6715603

[6] Cui C, Wang Y, Liu J, Zhao J, Sun P, Wang S. A fungal pathogen deploys a small silencing RNA that attenuates mosquito immunity and facilitates infection. Nat Commun. 2019;10: 4298 10.1038/s41467-019-12323-1 31541102 PMC6754459

[7] Shahid S, Kim G, Johnson NR, Wafula E, Wang F, Coruh C, et al MicroRNAs from the parasitic plant *Cuscuta campestris* target host messenger RNAs. Nature. 2018;553: 82–85. 10.1038/nature25027 29300014

[8] Ren B, Wang X, Duan J, Ma J. Rhizobial tRNA-derived small RNAs are signal molecules regulating plant nodulation. Science. 2019;365: 919–922. 10.1126/science.aav8907 31346137

[9] Shimura H, Pantaleo V, Ishihara T, Myojo N, Inaba J, Sueda K, et al A viral satellite RNA induces yellow symptoms on tobacco by targeting a gene involved in chlorophyll biosynthesis using the RNA silencing machinery. PLoS Pathog. 2011;7: e1002021 10.1371/journal.ppat.1002021 21573143 PMC3088725

[10] Smith NA, Eamens AL, Wang MB. Viral small interfering RNAs target host genes to mediate disease symptoms in plants. PLoS Pathog. 2011;7: e1002022 10.1371/journal.ppat.1002022 21573142 PMC3088724

[11] Adkar-Purushothama CR, Brosseau C, Giguere T, Sano T, Moffett P, Perreault JP. Small RNA derived from the virulence modulating region of the *Potato spindle tuber viroid* silences *callose synthase* genes of tomato plants. Plant Cell. 2015;27: 2178–2194. 10.1105/tpc.15.00523 26290537 PMC4568511

[12] Grey F, Tirabassi R, Meyers H, Wu GM, McWeeney S, Hook L, et al A viral microRNA down-regulates multiple cell cycle genes through mRNA 5 ' UTRs. Plos Pathog. 2010;6: e1000967 10.1371/journal.ppat.1000967 20585629 PMC2891821

[13] Pfeffer S, Zavolan M, Grasser FA, Chien MC, Russo JJ, Ju JY, et al Identification of virus-encoded microRNAs. Science. 2004;304: 734–736. 10.1126/science.1096781 15118162

[14] Pfeffer S. Viral miRNAs: tiny but mighty helpers for large and small DNA viruses. Future Virol. 2008;3: 291–299.

[15] Yang Y, Liu T, Shen D, Wang J, Ling X, Hu Z, et al Tomato yellow leaf curl virus intergenic siRNAs target a host long noncoding RNA to modulate disease symptoms. PLoS Pathog. 2019;15: e1007534 10.1371/journal.ppat.1007534 30668603 PMC6366713

[16] Cai Q, Qiao L, Wang M, He B, Lin FM, Palmquist J, et al Plants send small RNAs in extracellular vesicles to fungal pathogen to silence virulence genes. Science. 2018;360: 1126–1129. 10.1126/science.aar4142 29773668 PMC6442475

[17] Cai Q, Jin H. Small RNA extraction and quantification of isolated fungal cells from plant tissue by the sequential protoplastation Methods in Molecular Biology on “RNA Abundance Analysis”, second edition, Springer Protocols In press. 2019. 10.1007/978-1-0716-0743-5_16 32797462

[18] Zhang T, Zhao YL, Zhao JH, Wang S, Jin Y, Chen ZQ, et al Cotton plants export microRNAs to inhibit virulence gene expression in a fungal pathogen. Nat Plants. 2016;2: 16153 10.1038/nplants.2016.153 27668926

[19] Jiao J, Peng D. Wheat microRNA1023 suppresses invasion of *Fusarium graminearum* via targeting and silencing *FGSG_03101*. J Plant Interact. 2018;13: 514–521.

[20] Hou YN, Zhai Y, Feng L, Karimi HZ, Rutter BD, Zeng LP, et al A *Phytophthora* effector suppresses trans-kingdom RNAi to promote disease susceptibility. Cell Host Microbe. 2019;25: 153–165. 10.1016/j.chom.2018.11.007 30595554 PMC9208300

[21] Buck AH, Coakley G, Simbari F, McSorley HJ, Quintana JF, Le Bihan T, et al Exosomes secreted by nematode parasites transfer small RNAs to mammalian cells and modulate innate immunity. Nat Commun. 2014;5: 5488 10.1038/ncomms6488 25421927 PMC4263141

[22] LaMonte G, Philip N, Reardon J, Lacsina JR, Majoros W, Chapman L, et al Translocation of sickle cell erythrocyte microRNAs into *Plasmodium falciparum* inhibits parasite translation and contributes to malaria resistance. Cell Host Microbe. 2012;12: 187–199. 10.1016/j.chom.2012.06.007 22901539 PMC3442262

[23] Wang Z, Xi J, Hao X, Deng W, Liu J, Wei C, et al Red blood cells release microparticles containing human argonaute 2 and miRNAs to target genes of *Plasmodium falciparum*. Emerg Microbes Infect. 2017;6: e75 10.1038/emi.2017.63 28831191 PMC5583671

[24] Liu SR, da Cunha AP, Rezende RM, Cialic R, Wei ZY, Bry L, et al The host shapes the gut microbiota via fecal microRNA. Cell Host Microbe. 2016;19: 32–43. 10.1016/j.chom.2015.12.005 26764595 PMC4847146

[25] Teng Y, Ren Y, Sayed M, Hu X, Lei C, Kumar A, et al Plant-derived exosomal microRNAs shape the gut microbiota. Cell Host Microbe. 2018;24: 637–652. 10.1016/j.chom.2018.10.001 30449315 PMC6746408

[26] Condon C, Putzer H. The phylogenetic distribution of bacterial ribonucleases. Nucleic Acids Res. 2002;30: 5339–5346. 10.1093/nar/gkf691 12490701 PMC140075

[27] Colombo M, Raposo G, Thery C. Biogenesis, secretion, and intercellular interactions of exosomes and other extracellular vesicles. Annu Rev Cell Dev Biol. 2014;30: 255–289. 10.1146/annurev-cellbio-101512-122326 25288114

[28] Andreu Z, Yanez-Mo M. Tetraspanins in extracellular vesicle formation and function. Front Immunol. 2014;5: 442 10.3389/fimmu.2014.00442 25278937 PMC4165315

[29] Valadi H, Ekstrom K, Bossios A, Sjostrand M, Lee JJ, Lotvall JO. Exosome-mediated transfer of mRNAs and microRNAs is a novel mechanism of genetic exchange between cells. Nat Cell Biol. 2007;9: 654–659. 10.1038/ncb1596 17486113

[30] D’Souza-Schorey C, Clancy JW. Tumor-derived microvesicles: shedding light on novel microenvironment modulators and prospective cancer biomarkers. Genes Dev. 2012;26: 1287–1299. 10.1101/gad.192351.112 22713869 PMC3387656

[31] Robbins PD, Morelli AE. Regulation of immune responses by extracellular vesicles. Nat Rev Immunol. 2014;14: 195–208. 10.1038/nri3622 24566916 PMC4350779

[32] Janas AM, Sapon K, Janas T, Stowell MHB, Janas T. Exosomes and other extracellular vesicles in neural cells and neurodegenerative diseases. Biochim Biophys Acta Biomembr. 2016;1858: 1139–1151. 10.1016/j.bbamem.2016.02.011 26874206

[33] Akers JC, Gonda D, Kim R, Carter BS, Chen CC. Biogenesis of extracellular vesicles (EV): exosomes, microvesicles, retrovirus-like vesicles, and apoptotic bodies. J Neurooncol. 2013;113: 1–11. 10.1007/s11060-013-1084-8 23456661 PMC5533094

[34] Crescitelli R, Lasser C, Szabo TG, Kittel A, Eldh M, Dianzani I, et al Distinct RNA profiles in subpopulations of extracellular vesicles: apoptotic bodies, microvesicles and exosomes. J Extracell Vesicles. 2013;2. 10.3402/jev.v2i0.20677 PMC3823106 24223256

[35] Bergsmedh A, Szeles A, Henriksson M, Bratt A, Folkman MJ, Spetz AL, et al Horizontal transfer of oncogenes by uptake of apoptotic bodies. Proc Natl Acad Sci U S A. 2001;98: 6407–6411. 10.1073/pnas.101129998 11353826 PMC33481

[36] Zernecke A, Bidzhekov K, Noels H, Shagdarsuren E, Gan L, Denecke B, et al Delivery of microRNA-126 by apoptotic bodies induces CXCL12-dependent vascular protection. Sci Signal. 2009;2: ra81 10.1126/scisignal.2000610 19996457

[37] Coakley G, Maizels RM, Buck AH. Exosomes and other extracellular vesicles: The new communicators in parasite infections. Trends Parasitol. 2015;31: 477–489. 10.1016/j.pt.2015.06.009 26433251 PMC4685040

[38] Chow FW, Koutsovoulos G, Ovando-Vazquez C, Neophytou K, Bermudez-Barrientos JR, Laetsch DR, et al Secretion of an Argonaute protein by a parasitic nematode and the evolution of its siRNA guides. Nucleic Acids Res. 2019;47: 3594–3606. 10.1093/nar/gkz142 30820541 PMC6468290

[39] Li J, Wu C, Wang W, He Y, Elkayam E, Joshua-Tor L, et al Structurally modulated codelivery of siRNA and Argonaute 2 for enhanced RNA interference. Proc Natl Acad Sci U S A. 2018;115: E2696–E2705. 10.1073/pnas.1719565115 29432194 PMC5866582

[40] Eichenberger RM, Ryan S, Jones L, Buitrago G, Polster R, Montes de Oca M, et al Hookworm secreted extracellular vesicles interact with host cells and prevent inducible colitis in mice. Front Immunol. 2018;9: 850 10.3389/fimmu.2018.00850 29760697 PMC5936971

[41] Roig J, Saiz ML, Galiano A, Trelis M, Cantalapiedra F, Monteagudo C, et al Extracellular vesicles from the helminth *Fasciola hepatica* prevent DSS-Induced acute ulcerative colitis in a T-lymphocyte independent mode. Front Microbiol. 2018;9: 1036 10.3389/fmicb.2018.01036 29875750 PMC5974114

[42] Halperin W, Jensen WA. Ultrastructural changes during growth and embryogenesis in carrot cell cultures. J Ultrastruct Res. 1967;18: 428–443. 10.1016/s0022-5320(67)80128-x 6025110

[43] Regente M, Corti-Monzon G, Maldonado AM, Pinedo M, Jorrin J, de la Canal L. Vesicular fractions of sunflower apoplastic fluids are associated with potential exosome marker proteins. FEBS Lett. 2009;583: 3363–3366. 10.1016/j.febslet.2009.09.041 19796642

[44] Boavida LC, Qin P, Broz M, Becker JD, McCormick S. Arabidopsis tetraspanins are confined to discrete expression domains and cell types in reproductive tissues and form homo- and heterodimers when expressed in yeast. Plant Physiol. 2013;163: 696–712. 10.1104/pp.113.216598 23946353 PMC3793051

[45] Rutter BD, Innes RW. Extracellular vesicles isolated from the leaf apoplast carry stress-response proteins. Plant Physiol. 2017;173: 728–741. 10.1104/pp.16.01253 27837092 PMC5210723

[46] Kwon C, Neu C, Pajonk S, Yun HS, Lipka U, Humphry M, et al Co-option of a default secretory pathway for plant immune responses. Nature. 2008;451: 835–840. 10.1038/nature06545 18273019

[47] Rutter BD, Rutter K. L. and Innes R. W. Isolation and quantification of plant extracellular vesicles. Bio-protocol. 2017;7: e2533. 10.21769/BioProtoc.2533 PMC8413617 34541189

[48] Baldrich P, Rutter BD, Zand Karimi H, Podicheti R, Meyers BC, Innes RW. Plant extracellular vesicles contain diverse small RNA species and are enriched in 10 to 17 nucleotide "Tiny" RNAs. Plant Cell. 2019;31: 315–324. 10.1105/tpc.18.00872 30705133 PMC6447009

[49] Meyer D, Pajonk S, Micali C, O’Connell R, Schulze-Lefert P. Extracellular transport and integration of plant secretory proteins into pathogen-induced cell wall compartments. Plant J. 2009;57: 986–999. 10.1111/j.1365-313X.2008.03743.x 19000165

[50] Rutter BD, Innes RW. Extracellular vesicles as key mediators of plant-microbe interactions. Curr Opin Plant Biol. 2018;44: 16–22. 10.1016/j.pbi.2018.01.008 29452903

[51] Wang J, Ding Y, Wang J, Hillmer S, Miao Y, Lo SW, et al EXPO, an exocyst-positive organelle distinct from multivesicular endosomes and autophagosomes, mediates cytosol to cell wall exocytosis in Arabidopsis and tobacco cells. Plant Cell. 2010;22: 4009–4030. 10.1105/tpc.110.080697 21193573 PMC3027174

[52] Willms E, Cabanas C, Mager I, Wood MJA, Vader P. Extracellular vesicle heterogeneity: Subpopulations, isolation techniques, and diverse functions in cancer progression. Front Immunol. 2018;9: 738 10.3389/fimmu.2018.00738 29760691 PMC5936763

[53] Arroyo JD, Chevillet JR, Kroh EM, Ruf IK, Pritchard CC, Gibson DF, et al Argonaute2 complexes carry a population of circulating microRNAs independent of vesicles in human plasma. Proc Natl Acad Sci U S A. 2011;108: 5003–5008. 10.1073/pnas.1019055108 21383194 PMC3064324

[54] Michell DL, Allen RM, Landstreet SR, Zhao SL, Toth CL, Sheng QH, et al Isolation of high-density lipoproteins for non-coding small RNA quantification. J Vis Exp. 2016;28: 54488. 10.3791/54488 PMC5226318 27929461

[55] Vickers KC, Palmisano BT, Shoucri BM, Shamburek RD, Remaley AT. MicroRNAs are transported in plasma and delivered to recipient cells by high-density lipoproteins. Nat Cell Biol. 2011;13: 423–433. 10.1038/ncb2210 21423178 PMC3074610

[56] Turchinovich A, Weiz L, Langheinz A, Burwinkel B. Characterization of extracellular circulating microRNA. Nucleic Acids Res. 2011;39: 7223–7233. 10.1093/nar/gkr254 21609964 PMC3167594

[57] Zhang Q, Higginbotham JN, Jeppesen DK, Yang YP, Li W, McKinley ET, et al Transfer of functional cargo in exomeres. Cell Rep. 2019;27: 940–954. 10.1016/j.celrep.2019.01.009 30956133 PMC6559347

[58] Zhang HY, Freitas D, Kim HS, Fabijanic K, Li Z, Chen HY, et al Identification of distinct nanoparticles and subsets of extracellular vesicles by asymmetric flow field-flow fractionation. Nat Cell Biol. 2018;20: 332–343. 10.1038/s41556-018-0040-4 29459780 PMC5931706

[59] Thery C, Amigorena S, Raposo G, Clayton A. Isolation and characterization of exosomes from cell culture supernatants and biological fluids. Curr Protoc Cell Biol. 2006;Chapter 3: Unit 3 22 10.1002/0471143030.cb0322s30 18228490

[60] Jeppesen DK, Fenix AM, Franklin JL, Higginbotham JN, Zhang Q, Zimmerman LJ, et al Reassessment of exosome composition. Cell. 2019;177: 428–445 e18. 10.1016/j.cell.2019.02.029 30951670 PMC6664447

[61] Kowal J, Arras G, Colombo M, Jouve M, Morath JP, Primdal-Bengtson B, et al Proteomic comparison defines novel markers to characterize heterogeneous populations of extracellular vesicle subtypes. Proc Natl Acad Sci U S A. 2016;113: E968–E977. 10.1073/pnas.1521230113 26858453 PMC4776515

[62] Rodrigues ML, Nimrichter L, Oliveira DL, Frases S, Miranda K, Zaragoza O, et al Vesicular polysaccharide export in *Cryptococcus neoformans* is a eukaryotic solution to the problem of fungal trans-cell wall transport. Eukaryot Cell. 2007;6: 48–59. 10.1128/EC.00318-06 17114598 PMC1800364

[63] Peres da Silva R, Puccia R, Rodrigues ML, Oliveira DL, Joffe LS, Cesar GV, et al Extracellular vesicle-mediated export of fungal RNA. Sci Rep. 2015;5: 7763 10.1038/srep07763 25586039 PMC5379013

[64] Fisher MC, Hawkins NJ, Sanglard D, Gurr SJ. Worldwide emergence of resistance to antifungal drugs challenges human health and food security. Science. 2018;360: 739–742. 10.1126/science.aap7999 29773744

[65] Nunes CC, Dean RA. Host-induced gene silencing: a tool for understanding fungal host interaction and for developing novel disease control strategies. Mol Plant Pathol. 2012;13: 519–529. 10.1111/j.1364-3703.2011.00766.x 22111693 PMC6638818

[66] Nowara D, Gay A, Lacomme C, Shaw J, Ridout C, Douchkov D, et al HIGS: host-induced gene silencing in the obligate biotrophic fungal pathogen *Blumeria graminis*. Plant Cell. 2010;22: 3130–3141. 10.1105/tpc.110.077040 20884801 PMC2965548

[67] Whangbo JS, Hunter CP. Environmental RNA interference. Trends Genet. 2008;24: 297–305. 10.1016/j.tig.2008.03.007 18450316

[68] Shih JD, Hunter CP. SID-1 is a dsRNA-selective dsRNA-gated channel. RNA. 2011;17: 1057–1065. 10.1261/rna.2596511 21474576 PMC3096038

[69] McEwan DL, Weisman AS, Huntert CP. Uptake of extracellular double-stranded RNA by SID-2. Mol Cell. 2012;47: 746–754. 10.1016/j.molcel.2012.07.014 22902558 PMC3488460

[70] Bolognesi R, Ramaseshadri P, Anderson J, Bachman P, Clinton W, Flannagan R, et al Characterizing the mechanism of action of double-stranded RNA activity against western corn rootworm (*Diabrotica virgifera virgifera* LeConte). Plos One. 2012;7: e47534 10.1371/journal.pone.0047534 23071820 PMC3469495

[71] Winston WM, Sutherlin M, Wright AJ, Feinberg EH, Hunter CP. *Caenorhabditis elegans* SID-2 is required for environmental RNA interference. Proc Natl Acad Sci U S A. 2007;104: 10565–10570. 10.1073/pnas.0611282104 17563372 PMC1965553

[72] Cai Q, He B, Kogel KH, Jin H. Cross-kingdom RNA trafficking and environmental RNAi-nature’s blueprint for modern crop protection strategies. Curr Opin Microbiol. 2018;46: 58–64. 10.1016/j.mib.2018.02.003 29549797 PMC6499079

[73] Koch A, Biedenkopf D, Furch A, Weber L, Rossbach O, Abdellatef E, et al An RNAi-based control of *Fusarium graminearum* infections through spraying of long dsRNAs involves a plant passage and is controlled by the fungal silencing machinery. Plos Pathog. 2016;12: e1005901 10.1371/journal.ppat.1005901 27737019 PMC5063301

[74] McLoughlin AG, Wytinck N, Walker PL, Girard IJ, Rashid KY, de Kievit T, et al Identification and application of exogenous dsRNA confers plant protection against *Sclerotinia sclerotiorum* and *Botrytis cinerea*. Sci Rep. 2018;8: 7320 10.1038/s41598-018-25434-4 29743510 PMC5943259

[75] Mitter N, Worrall EA, Robinson KE, Li P, Jain RG, Taochy C, et al Clay nanosheets for topical delivery of RNAi for sustained protection against plant viruses. Nat Plants. 2017;3: 16207 10.1038/nplants.2016.207 28067898

[76] Regente M, Pinedo M, San Clemente H, Balliau T, Jamet E, de la Canal L. Plant extracellular vesicles are incorporated by a fungal pathogen and inhibit its growth. J Exp Bot. 2017;68: 5485–5495. 10.1093/jxb/erx355 29145622

[77] Tatiparti K, Sau S, Kashaw SK, Iyer AK. siRNA delivery strategies: A comprehensive review of recent developments. Nanomaterials (Basel). 2017;7: E77. 28379201 10.3390/nano7040077 PMC5408169

[78] Groll AH, Rijnders BJA, Walsh TJ, Adler-Moore J, Lewis RE, Bruggemann RJM. Clinical pharmacokinetics, pharmacodynamics, safety and efficacy of liposomal amphotericin B. Clin Infect Dis. 2019;68: S260–S274. 10.1093/cid/ciz076 31222253 PMC6495018

[79] Walker L, Sood P, Lenardon MD. The viscoelastic properties of the fungal cell wall allow traffic of AmBisome as intact liposome vesicles. mBio. 2018;9: e02383–17. 10.1128/mBio.02383-17 29437927 PMC5801470

[80] Mullard A. FDA approves landmark RNAi drug. Nat Rev Drug Discov. 2018;17: 613. 10.1038/nrd.2018.152 30160250

[81] Garcia-Solache MA, Casadevall A. Global warming will bring new fungal diseases for mammals. Mbio. 2010;1: e00061–10. 10.1128/mBio.00061-10 20689745 PMC2912667

[82] Srinivasan S, Yeri A, Cheah PS, Chung A, Danielson K, De Hoff P, et al Small RNA sequencing across diverse biofluids identifies optimal methods for exRNA isolation. Cell. 2019;177: 446–462. 10.1016/j.cell.2019.03.024 30951671 PMC6557167

[83] Murillo OD, Thistlethwaite W, Rozowsky J, Subramanian SL, Lucero R, Shah N, et al exRNA Atlas analysis reveals distinct extracellular RNA cargo types and their carriers present across human biofluids. Cell. 2019;177: 463–477 10.1016/j.cell.2019.02.018 30951672 PMC6616370

[84] Das S, Ansel KM, Bitzer M, Breakefield XO, Charest A, Galas DJ, et al The extracellular RNA communication consortium: Establishing foundational knowledge and technologies for extracellular RNA research. Cell. 2019;177: 231–242. 10.1016/j.cell.2019.03.023 30951667 PMC6601620

[85] Shurtleff MJ, Temoche-Diaz MM, Karfilis KV, Ri S, Schekman R. Y-box protein 1 is required to sort microRNAs into exosomes in cells and in a cell-free reaction. Elife. 2016;5: e19276 10.7554/eLife.19276 27559612 PMC5047747

